# A high urea-to-creatinine ratio predicts long-term mortality independent of acute kidney injury among patients hospitalized with an infection

**DOI:** 10.1038/s41598-020-72815-9

**Published:** 2020-09-24

**Authors:** Elisabeth C. van der Slikke, Bastiaan S. Star, Vincent D. de Jager, Marije B. M. Leferink, Lotte M. Klein, Vincent M. Quinten, Tycho J. Olgers, Jan C. ter Maaten, Hjalmar R. Bouma

**Affiliations:** 1 Department of Clinical Pharmacy and Pharmacology, University Medical Center Groningen, University of Groningen, P.O. Box 30.001, 9700 RB Groningen, The Netherlands; 2 Department of Internal Medicine, Section of Acute Medicine, University Medical Center Groningen, University of Groningen, Groningen, The Netherlands; 3 Department of Internal Medicine, Evangelisches Krankenhaus Oldenburg, Oldenburg, Germany

**Keywords:** Biomarkers, Kidney, Kidney diseases, Infection

## Abstract

Acute kidney injury (AKI) occurs frequently in patients with sepsis. Persistent AKI is, in contrast to transient AKI, associated with reduced long-term survival after sepsis, while the effect of AKI on survival after non-septic infections remains unknown. As prerenal azotaemia is a common cause of transient AKI that might be identified by an increased urea-to-creatinine ratio, we hypothesized that the urea-to-creatinine ratio may predict the course of AKI with relevance to long-term mortality risk. We studied the association between the urea-to-creatinine ratio, AKI and long-term mortality among 665 patients presented with an infection to the ED with known pre-existent renal function. Long-term survival was reduced in patients with persistent AKI. The urea-to-creatinine ratio was not associated with the incidence of either transient or non-recovered AKI. In contrast, stratification according to the urea-to-creatinine-ratio identifies a group of patients with a similar long-term mortality risk as patients with persistent AKI. Non-recovered AKI is strongly associated with all-cause long-term mortality after hospitalization for an infection. The urea-to-creatinine ratio should not be employed to predict prerenal azotaemia, but identifies a group of patients that is at increased risk for long-term mortality after infections, independent of AKI and sepsis.

## Introduction

Sepsis is a life-threatening condition accompanied by organ dysfunction subsequent to a dysregulated host response to infection^1^. In the last decade, the incidences of sepsis and severe sepsis were approximately 437 and 270 per 100,000 person-years in high-income countries, while the in-hospital mortality rates of sepsis and severe sepsis were as high as 17% and 26%, respectively^1^. The incidence of severe infections and sepsis have more than doubled between 2000 and 2007, and is expected to keep rising due to increased lifespan, chronic diseases, use of immunosuppressant therapies, chemotherapy and invasive procedures^2,3^. Furthermore, patients surviving an initial septic episode in the hospital have a decreased life expectancy and reduced quality of life^4,5^. Thus, assessment of short-term outcome in sepsis might underestimate the total impact of sepsis. Yet, most studies focus on short-term outcome in sepsis, resulting in a lack of insight in early predictors of long-term outcome after sepsis. The occurrence of acute kidney injury (AKI) strongly affects long-term outcome after sepsis. Up to 60% of patients with sepsis develops AKI^6,7^, which is associated with failure of other organ systems, increased in-hospital mortality rate^6,8,9^, and development of chronic kidney disease (CKD) after hospital discharge both with transient (i.e. AKI that is recovered at discharge) and non-recovered AKI^10,11^. The occurrence of sepsis-AKI is strongly associated with an increased risk for long-term mortality after hospital discharge if not recovered, while *transient* AKI does not affect long-term mortality^9,10,12^.

AKI also occurs in up to 25% of patients with non-severe infections presenting to the emergency department (ED)^13,14^. Yet, it is not known whether (non-recovered) AKI is a similar risk factor for long-term mortality after a less severe infection as is the case in sepsis. Prerenal azotaemia is a common cause of transient AKI in patients with infection that is usually reversed by adequate fluid replacement within 24–72 h^15^. An increased urea-to-creatinine ratio might indicate a prerenal azotaemia as cause of AKI, as urea is reabsorbed with water during hypovolemia^16–18^. Consequently, an increased urea-to-creatinine ratio may identify patients with *transient* AKI that are not at risk for long-term mortality after sepsis and potentially also among patients with less severe infections. In other patients, such as patients with heart failure or undergoing dialysis, an increased urea-to-creatinine ratio is already associated with all-cause long-term mortality^19–21^. The association between the urea-to-creatinine ratio and the course of both types of AKI with long-term mortality with AKI in patients with infection is not yet known. This information could be used for risk stratification in patients with an infection or sepsis and might be of prognostic importance, specifically for patients with infection without signs of sepsis where the SOFA score cannot be used to estimate outcome. In this study, we investigated this association among patients presenting to the ED with an infection, either with or without signs of sepsis. Although AKI that is not recovered at hospital discharge is associated with increased long-term mortality risk^9,10,12^, the course of renal function is not known upon admission to the emergency department. Defining the urea-to-creatinine ratio may identify patients at increased risk for long-term mortality already upon presentation to the emergency department, when the course of AKI is not yet known.

## Methods

### Study design and setting

This is a prospective observational study of adults patients visiting the emergency department (ED) of the University Medical Center Groningen (UMCG), the Netherlands between March 2016 and April 2018. The protocol was reviewed by the ethical review board of the UMCG and approved by a waiver (METc 2015/164). Written informed consent was obtained from all participating patients. The study adhered to the Strengthening the Reporting of Observational Studies in Epidemiology (STROBE) recommendation for cohort studies^22^.

### Study population

Adult patients $(\ge$ 18 years of age) visiting the ED of the UMCG who presented with a suspected infection (as determined by the treating physician upon initial contact based on focal symptoms suggestive of an infection [e.g. productive cough, dyspnoea, dysuria, pollakisuria, abdominal pain, erythema]) and/or fever (≥ 38 °C, either at home or upon triage in the ED) between 8:00–21:00 h were included in the UMCG sepsis database (*n* = 914). If the initial workup at the ED demonstrated a non-infectious cause of the signs and symptoms, patients were not included. We excluded 99 re-visits, 57 patients who did not have an infection and 48 patients who died within 28 days (short-term) after presentation to the ED. Next, 7 patients on dialysis before presentation to the ED, 57 patients without known pre-existent serum creatinine function, 10 patients in whom the kidney function was not measured at the ED and 3 patients who started dialysis during hospitalization were also excluded from further analysis, leaving 665 patients in this study.

### Data collection

Collected data included demographic characteristics, vital parameters and laboratory measurements at admission, as well as during hospitalization. Patient characteristics consisted of age, sex, history of diabetes mellitus, chronic kidney disease, kidney transplantation, cardiovascular disease (defined as chronic heart failure and/or ischemic heart disease) and active cancer (defined as radiotherapy or chemotherapy treatment received up to two years prior to the current hospitalization). Data was collected from the electronic patient files and in case the electronic patient file did not contain the necessary data, in addition by interviewing patients and physicians. Pre-existing kidney function, defined as the most recent creatinine and/or urea level that was measured within the year prior to presentation, was collected retrospectively from the electronic patient files. AKI was defined according to the KDIGO criteria as an increase of at least 50% of serum creatinine upon admission or an absolute rise of more than 26.4 µmol/L within 48 h after admission as compared to pre-existing serum creatinine^12,13^. Transient AKI was defined as presence of AKI at the ED that was recovered at discharge (defined as a decrease in serum creatinine level to less than 50% or 26.4 µmol/L above baseline), while non-recovered AKI (i.e. persistent or non-transient AKI) was defined as presence of AKI at the ED which had not recovered at discharge (i.e. serum creatinine at hospital discharge at least 50% or 26.4 µmol/L higher as compared to baseline). As data collection started prior to the introduction of the Sepsis-3 criteria, sepsis was defined based on the Sepsis-2 criteria: a suspected or confirmed infection in the presence of two or more systemic inflammatory response syndrome (SIRS) criteria; (1) body temperature > 38 °C or < 36 °C, (2) heart frequency > 90 beats/min, (3) respiratory rate > 20 breaths/min or PaCO_2_ < 32 mmHg, and (4) white blood cell count > 12,000 cells/mm^3^, < 4,000 cells/mm^3^ or > 10% immature (band) forms^23^. Septic shock was defined as persistent hypotension (systolic blood pressure < 90 mmHg) after at least 2 L of intravenous fluid in patients with sepsis. The serum urea-to-creatinine ratio at presentation was calculated as follows: serum urea (mmol/L)/serum creatine (µmol/L). To convert the presented SI-units to conventional units (i.e. BUN and serum creatinine in mg/dL) the presented ratio should be divided by four (i.e. multiply urea by 2.80, multiply creatinine by 11.13). To determine whether a rise in the urea-to-creatinine ratio, which is suggestive of pre-renal azotaemia, might allow early identification of patients with transient AKI already upon presentation to the ED, we stratified patients into tertiles based on their urea-to-creatinine ratio.

### Outcome

The primary outcome of this study was all-cause mortality during long-term follow-up (i.e. at least 28 days after presentation to the ED until the end of follow-up (maximum 3 years). Information regarding the mortality status during follow-up was obtained from the Municipal Personal Records Database, which contains a complete overview of mortality of all residents in the Netherlands. The Municipal Personal Records Database is a national database and consequently, moving to another city will not affect mortality registration.

### Data analysis

Statistical analyses were performed using SPSS 23 for Windows (IBM, USA). Patient demographics were analysed using a Chi-Square test for categorical variables, and Mann–Whitney U followed by a Kruskal–Wallis test for continuous variables, as most were not normally distributed. Kaplan–Meier survival curves were created with GraphPad Prism 7 (GraphPad Software, USA), while differences between groups were analysed by a Mantel-Cox log rank test. Since levels of serum urea and creatinine might be affected by medication use, we correlated the defined daily dose (DDD) of ACEi/ARBs and diuretics with serum urea and creatinine levels using a Pearson’s correlation test (two-tailed). The association of urea-to-creatinine ratio, age, sex, co-morbidity, drug use and sepsis severity with AKI was calculated using a univariable binary logistic regression analysis (cut-off *p* < 0.10), followed by a multivariable binary logistic regression analysis (forward: conditional, *p*-entry 0.05, *p*-removal 0.10). Further, univariable Cox-regression analysis (cut-off *p* < 0.10), followed by a multivariable Cox regression analysis to calculate the associations between AKI, urea-to-creatinine ratio, age, sex, co-morbidity, drug use and sepsis severity with long-term all-cause mortality.

### Statement of methods used

All methods were carried out in accordance with relevant guidelines and regulations.

### Statement of approval

The protocol was reviewed by the ethical review board of the UMCG and approved by a waiver (METc 2015/164). Written informed consent was obtained from all participating patients.

## Results

### Long-term all-cause mortality rate is higher in patients with non-recovered AKI

**Figure 1:**
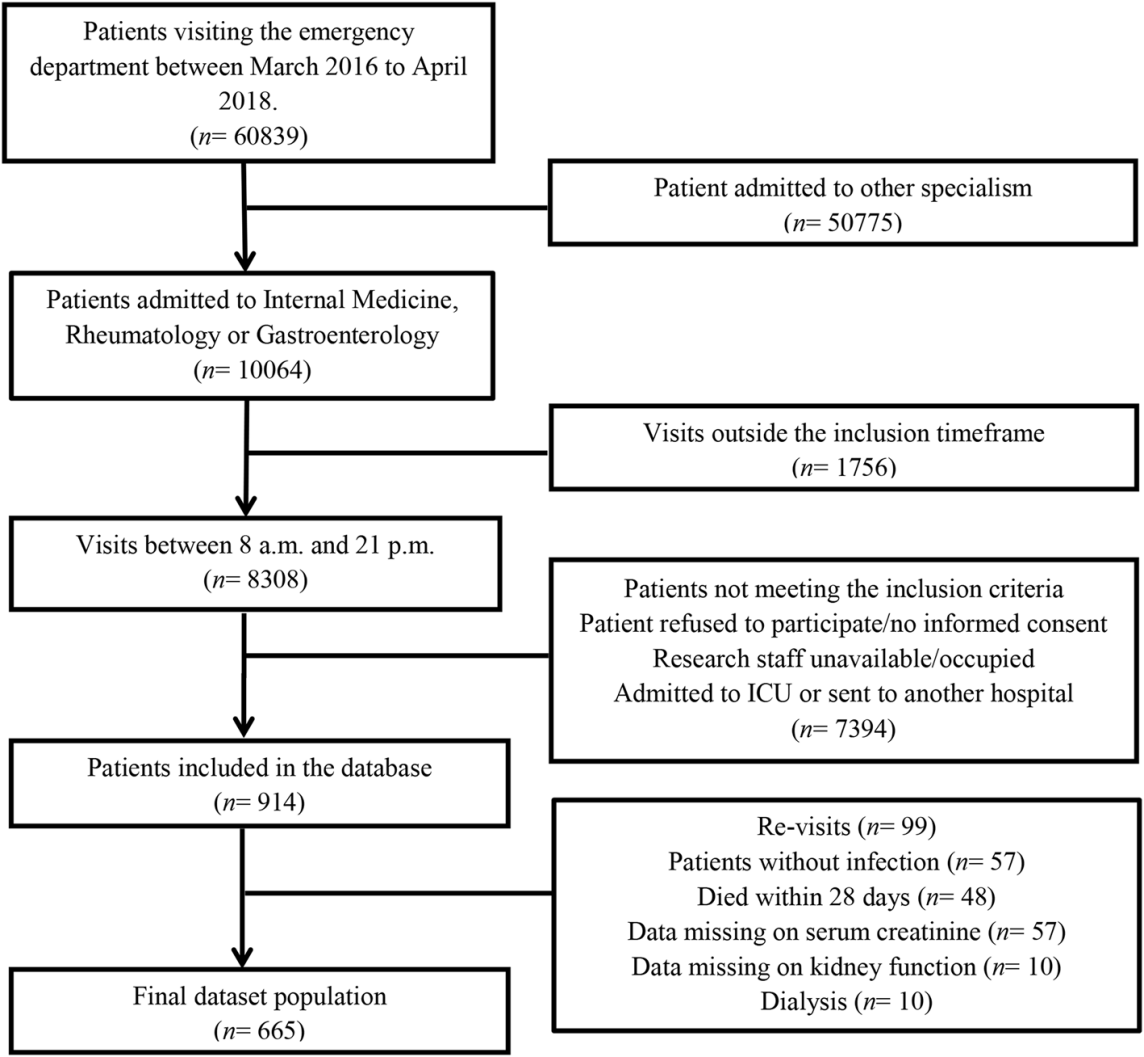
Flow chart of patient selection. Adult medical patients visiting the emergency department of the UMCG between March 2016 and April 2018 were screened for inclusion.

**Figure 2:**
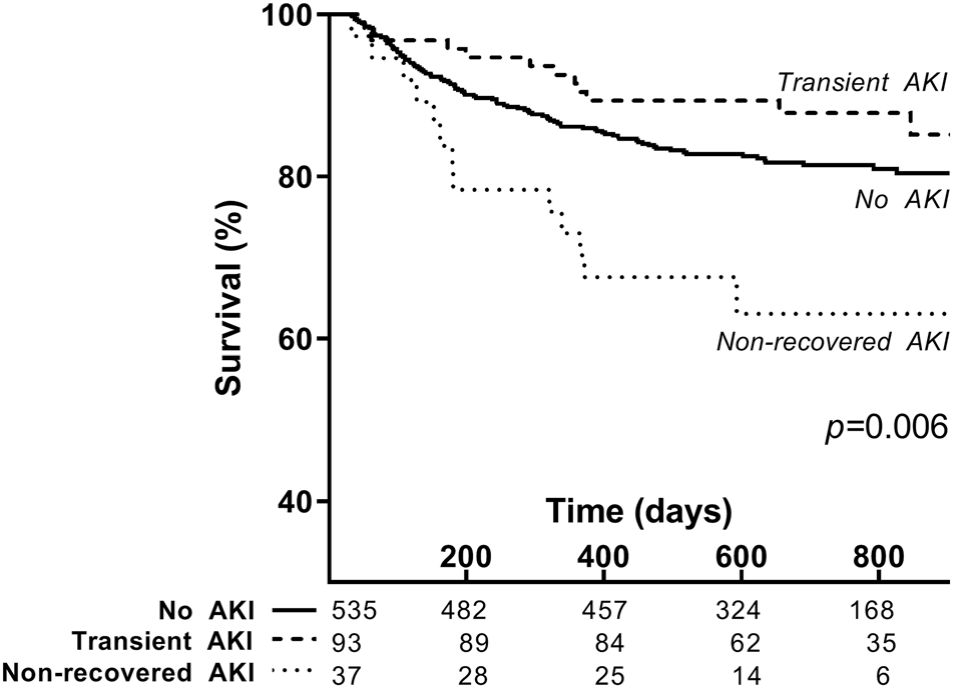
Survival of patients with and without acute kidney injury. Kaplan–Meier survival curve for patients suffering from infection without AKI, with transient AKI or with non-recovered AKI. *AKI* acute kidney injury.

We studied the effect of acute kidney injury and urea-to-creatinine ratio on long-term mortality in 665 patients presented with an infection to the ED and who were alive at the moment of hospital discharge (Fig. 1). The median SIRS score was 2 (2–3, 95% CI) and 476 (72%) patients had sepsis, based on the Sepsis-2 criteria. In total 14 (2%) had septic shock, while 21 patients (3%) were admitted to the intensive care unit (ICU). During the median follow-up duration of 686 days (664–713, 95% CI) after presentation to the ED, in total 123 (18%) patients died (Table 1). More than half of the patients had a heart frequency of more than 90 beats per minute (54%) and one third had a respiratory frequency of more than 20 per minute (34%), while other SIRS criteria were met less frequently (Table 1). The most common infection focus was in the respiratory tract (31%), followed by urogenital (19%) and abdominal infections (17%) (Table 1). Although patients might have more than one diagnosis upon presentation to the ED, none of the patients had a gastro-intestinal bleeding, which are known to increase the urea-to-creatinine-ratio as well. AKI was present in 130 patients (19%), which was recovered at hospital discharge in the vast majority (*n* = 93, 72%) (Table 1). In order to assess the association between transient versus recovered AKI and long-term all-cause mortality, we generated Kaplan–Meier survival curves of the patients stratified to type of AKI (Fig. 2). In contrast to transient AKI that was not associated with long-term mortality, non-recovered AKI was strongly associated with long-term all-cause mortality, as 13 of 37 (35%) patients with non-recovered AKI died during follow-up compared to 12 of 94 (13%) of patients who had recovered from AKI (*p* < 0.05, Fig. 2). Thus, in contrast to transient AKI, non-recovered AKI, which occurred in 6% of the patients presenting with an infection to the ED, was associated with increased long-term all-cause mortality.

**Table 1:** Demographic and disease characteristics of patients.

	All patients^a^ (*n* = 665)	Urea-to-creatinine ratio		
		< 61 (*n* = 221)	61–84 (*n* = 223)	> 84 (*n* = 221)
Age (years)	63 (62–65)	56 (54–60) ^A^	63 (61–65) ^B^	66 (64–69) ^C^
Sex (male)	386 (58%)	130 (59%)	135 (61%)	119 (54%)
Co-morbidity				
Diabetes Mellitus	135 (20%)	39 (18%)	48 (22%)	46 (21%)
Chronic kidney disease	92 (14%)	40 (18%)	26 (11%)	26 (12%)
Kidney transplantation	93 (14%)	37 (17%)	29 (13%)	27 (12%)
Cardiovascular disease	146 (22%)	47 (21%)	41 (18%)	58 (26%)
Active cancer	212 (32%)	71 (32%)	67 (30%)	73 (33%)
Use of ACEi/ARB or diuretics				
Use of ACEi/ARB	157 (24%)	36 (17%) ^A^	58 (26%) ^B^	63 (28%) ^B^
Use of potassium-sparing diuretics	43 (6%)	8 (4%) ^A^	13 (6%) ^A,B^	22 (10%) ^B^
Use of thiazide diuretics	48 (7%)	9 (4%)	20 (9%)	19 (9%)
Use of loop diuretics	95 (14%)	20 (9%) ^A^	28 (13%) ^A^	47 (21%) ^B^
Use of NSAID	32 (5%)	11 (5%)	12 (5%)	9 (4%)
Systolic blood pressure (mmHg)	129 (125–130)	129 (125–131)	131 (126–132)^A^	126 (120–130)^B^
Diastolic blood pressure (mmHg)	77 (75–77)	77 (75–79)	78 (74–80)	75 (72–77)
SIRS score ≥ 2	476 (72%)	164 (74%)	156 (70%)	156 (71%)
SIRS criteria				
Temperature < 36 °C	16 (2%)	5 (2%)	2 (1%)	9 (4%)
Temperature > 38 °C	173 (26%)	52 (24%)	63 (28%)	58 (26%)
Heart frequency > 90 bpm	361 (54%)	121 (55%)	125 (56%)	115 (52%)
Respiratory frequency > 20/min	226 (34%)	62 (28%)	78 (35%)	86 (39%)
PaCO_2_ ≥ 4.3 kPa	150 (23%)	50 (23%)	44 (20%)	56 (25%)
Leukocytes < 4.0 × 10^9^/L	76 (11%)	33 (15%)	22 (10%)	21 (10%)
Leukocytes > 12.0 × 10^9^/L	212 (32%)	70 (32%)	79 (35%)	63 (29%)
Lactate (mmol/L)	1.3 (1.2–1.5)	1.2 (1.0–1.7)	1.2 (1.1–1.5)	1.4 (1.3–1.8)
CRP (mg/L)	67 (60–78)	62 (54–84)	66 (54–78)	79 (64–95)
Infection focus				
Respiratory tract	206 (31%)	62 (28%)	78 (35%)	66 (30%)
Urogenital	129 (19%)	49 (22%)	34 (15%)	46 (21%)
Soft tissue or joints	69 (10%)	16 (7%)	27 (12%)	26 (12%)
Abdominal	112 (17%)	39 (18%)	43 (19%)	30 (14%)
Central nervous system	9 (1%)	3 (1%)	2 (1%)	4 (2%)
Catheter-related	5 (1%)	3 (1%)	0 (0%)	2 (1%)
Other/unknown	137 (21%)	50 (23%)	39 (17%)	48 (22%)
Septic shock	14 (2%)	3 (1%)	6 (3%)	5 (2%)
Admitted to ICU	21 (3%)	8 (4%)	6 (3%)	5 (2%)
Deceased during follow-up	123 (18%)	26 (12%) ^A^	27 (12%) ^A^	70 (32%) ^B^
Follow-up duration (days)	686 (664–713)	729 (687–760) ^A^	700 (662–741) ^A^	605 (536–672) ^B^
Acute kidney injury				
No AKI	535 (81%)	177 (80%)	185 (83%)	174 (79%)
Recovered at discharge	93 (14%)	34 (15%)	28 (13%)	31 (14%)
Not recovered at discharge	37 (6%)	10 (5%)	10 (5%)	17 (8%)

*ACEi* ACE-inhibitor, *ARB* angiotensin receptor blocker, *NSAID* non-steroidal anti-inflammatory drug, *SIRS* systemic inflammatory response syndrome, *CRP* C-reactive protein, *ICU* intensive care unit, *AKI* acute kidney injury.

^a^Data are presented as *n* (%) for categorical variables or median (95% CI) for continuous variables. Different letters in superscript represent significant differences between groups (*p* < 0.05).

### An increased urea-to-creatinine ratio is not a predictor of transient AKI

To determine whether an increased urea-to-creatinine ratio upon admission to the ED is associated with recovery of AKI during hospitalization, patients were divided in tertiles according to their urea-to-creatinine ratio (< 61* n* = 221, 61–84 *n* = 223 and > 84 *n* = 221; Table 1). Patients with a higher urea-to-creatinine ratio were on average older, used more often ACE-inhibitors (ACEi), angiotensin receptor blockers (ARB), potassium-sparing diuretics and loop diuretics (Table 1). Co-morbidity and infection focus or severity (i.e. incidence of sepsis, septic shock, SIRS-score, and ICU admittance) was not different between the tertiles (Table 1). Patients with a high urea-to-creatinine-ratio presented with a significant rise in serum urea as compared to pre-existent values, while the serum creatinine levels were not significantly changed upon presentation to the ED as compared to baseline values (Table 2). Patients with the highest urea-to-creatinine-ratio had higher serum urea levels as compared to the other groups at baseline, hospital admission and discharge. Importantly, the incidence of transient AKI, non-recovered AKI and both types of AKI combined was not different between the different groups stratified according to the urea-to-creatinine levels (Table 1). Multivariate binary logistic regression analysis demonstrated that the urea-to-creatinine-ratio was not associated with recovery of renal function in the subgroup of patients who were admitted with AKI (*n* = 130, Table 3). In contrast, a higher SIRS score was associated with transient AKI (OR 1.55 [1.08–2.24, 95% CI], *p* = 0.02), while use of potassium-sparing diuretics (OR 0.27 [0.08–0.95, 95% CI], *p* = 0.04) or thiazide diuretics (OR 0.24 [0.08–0.75, 95% CI], *p* = 0.02) were associated with failure to recover from AKI (non-recovered AKI) (Table 3). In order to dissect which items of the SIRS score were associated with recovery from AKI, we entered the different SIRS criteria separately in the regression model. Of these, only heart rate > 90/min was associated with recovery of renal function (OR 3.06 [1.02–9.13, 95% CI], *p* = 0.04; constant OR 1.21, *p* = 0.59; model characteristics: *χ*^2^ 4.2, df 1, *p* < 0.05). Hence, an increased urea-to-creatinine ratio was not predictive of the course of AKI.

**Table 2:** Kidney function parameters.

	All patients^a^(*n* = 665)	Urea-to-creatinine ratio^b^		
		< 61 (*n* = 221)	61–84 (*n* = 223)	> 84 (*n* = 221)
Pre-existent kidney function				
Creatinine (µmol/L)	87 (84–91)	92 (87–97) ^A^	88 (82–95) ^B^	82 (77–88) ^B^
Urea (mmol/L)	6.4 (6.2–6.7)	5.6 (5.1–6.2) ^A^	6.1 (5.8–6.6) ^A^	7.3 (6.8–8.1) ^B^
Urea-to-creatinine ratio	71 (69–74)	57 (54–59) ^A^	69 (67–73) ^B^	89 (86–94) ^C^
Kidney function upon presentation				
Creatinine (µmol/L)	91 (88–95)	98 (91–109) ^A^	92 (85–97) ^B^	83 (79–92) ^B^
Relative change in creatinine	1.03 (1.01–1.05)	1.05 (1.00–1.07)	1.02 (1.00–1.05)	1.02 (1.00–1.05)
Urea (mmol/L)	6.9 (6.6–7.4)	5.0 (4.8–5.5) ^A^	6.6 (6.3–7.4) ^A^	8.8 (8.2–9.4) ^B^
Relative change in urea	1.05 (1.01–1.07)	0.91 (0.89–0.96) ^A^	1.07 (1.04–1.13) ^B^	1.15 (1.08–1.22) ^C^
Urea-to-creatinine ratio	72 (70–75)	53 (51–54) ^A^	72 (71–74) ^B^	103 (100–105) ^C^
Kidney function at discharge				
Creatinine (µmol/L)	83 (81–88)	89 (83–94) ^A^	84 (81–91) ^B^	79 (74–87) ^B^
Urea (mmol/L)	5.6 (5.4–6.1)	4.5 (4.3–5.0) ^A^	5.6 (5.4–6.4) ^B^	6.6 (6.4–7.4) ^B^
Urea-to-creatinine ratio	66 (63–68)	50 (49–52) ^A^	68 (66–70) ^B^	88 (85–93) ^C^

^a^Data are presented as *n* (%) for categorical variables or median (95%CI) for continuous variables. Different letters in superscript represent significant differences between groups (*p* < 0.05).

^b^The urea-to-creatinine ratio was calculated by dividing the serum urea (mmol/L) by the serum creatinine (µmol/L). To convert the presented urea-to-creatinine ratio in SI-units to conventional units (i.e. BUN and serum creatinine in mg/dL) divide the presented ratio by four.

**Table 3:** Multivariable binary logistic regression analysis of factors associated with acute kidney injury recovery in a subpopulation of patients with acute kidney injury.

	Adj. OR (95% CI)^a^	*p*-value
Constant	1.42	0.48
SIRS score	1.55 (1.08–2.24)	0.02
Potassium-sparing diuretics	0.27 (0.08–0.95)	0.04
Thiazide diuretics	0.24 (0.08–0.75)	0.01

*OR* Odds ratio, *95% CI* 95% Confidence interval, *SIRS* Systemic inflammatory response syndrome.

^a^In addition to the factors shown in the table, age, sex, co-morbidity (i.e. diabetes mellitus, cardiovascular disease, chronic kidney disease, malignancy), use of ACE-inhibitors, angiotensin receptor blocker, loop diuretics, thiazide diuretics or NSAID, septic shock, the relative change in creatinine and urea, and the urea-to-creatinine ratio upon admission as compared to pre-existing values were entered in the multivariable forward: conditional binary logistic regression analysis. Model characteristics: *χ*^2^ 14.1, df 3, *p* < 0.01.

### Patients with an increased urea-to-creatinine ratio are at increased risk for long-term all-cause mortality

**Figure 3:**
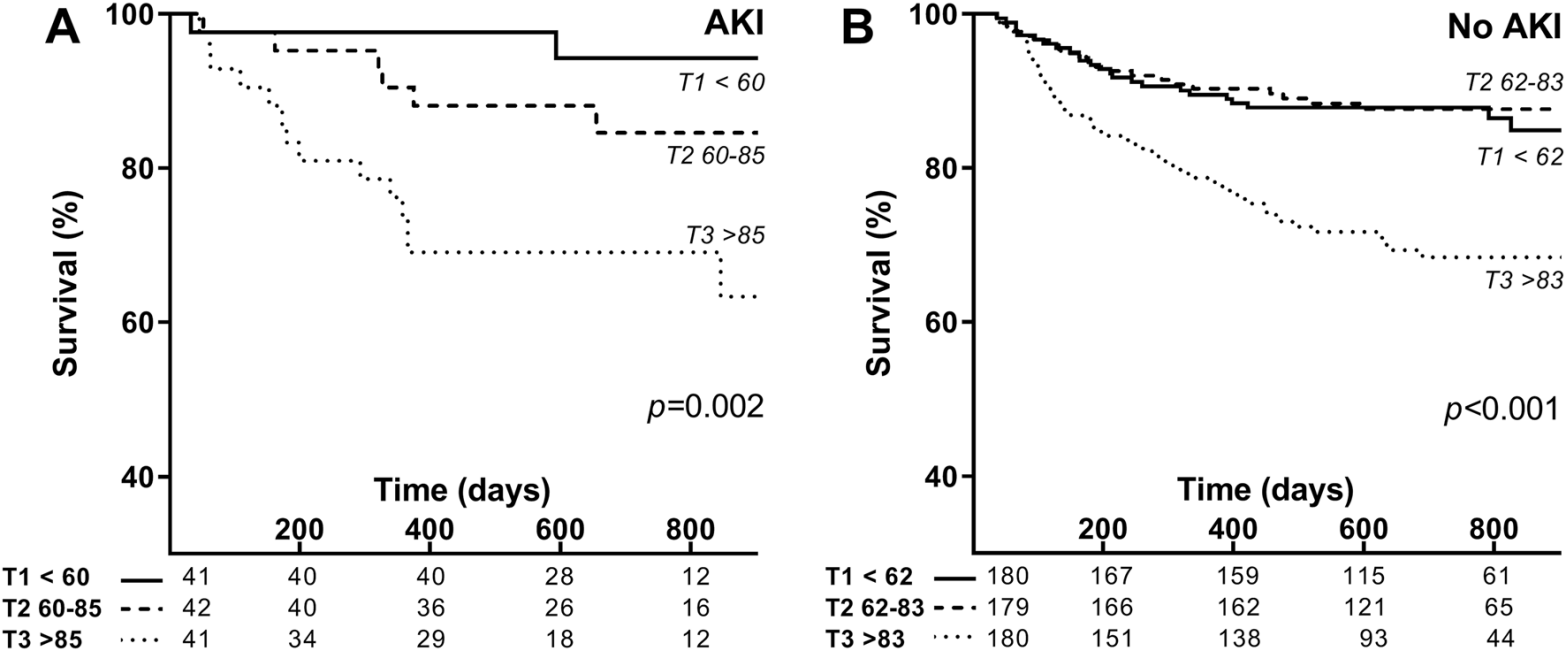
Urea-to-creatinine ratio and long-term mortality risk with and without acute kidney injury. Kaplan–Meier survival curve of the urea-to-creatinine ratio at hospital admission among patients with AKI (**A**) and without AKI (**B**). *AKI* acute kidney injury. The urea-to-creatinine ratio was calculated by dividing the serum urea (mmol/L) by the serum creatinine (µmol/L). To convert the presented urea-to-creatinine ratio in SI-units to conventional units (i.e. BUN and serum creatinine in mg/dL) divide the presented ratio by four.

To study whether an increased urea-to-creatinine ratio identifies patients at risk for long-term all-cause mortality, we stratified patients in tertiles according to the urea-to-creatinine ratio to generate Kaplan–Meier survival curves and perform a multivariate Cox regression survival analysis. The all-cause mortality rate was 32% among patients with the highest urea-to-creatinine ratio as compared to 12% among patients with the lowest urea-to-creatinine ratio during long-term follow-up of 605 (536–672) days and 729 (687–760) days, respectively (Table 1). An increased urea-to-creatinine ratio was associated with long-term all-cause mortality both among patients with and without AKI (*p* < 0.01, Fig. 3a,b). Also after adjusting the urea-to-creatinine ratio for non-recovered AKI, transient AKI, age, sex, co-morbidity (i.e. diabetes mellitus, cardiovascular disease, chronic kidney disease, malignancy), SIRS score, septic shock and the relative change in creatinine and urea as compared to baseline, the urea-to-creatinine ratio remained strongly and independently associated with long-term all-cause mortality (HR 2.00 [1.37–2.91, 95% CI], *p* < 0.01, Table 4). Other risk factors predictive of long-term all-cause mortality were age (HR 1.32 [1.18–1.47, 95% CI] per 10 years, *p* < 0.01), malignancy (HR 2.31 [1.61–3.32, 95% CI], *p* < 0.01) and non-recovered AKI (HR 1.86 [1.03–3.33, 95% CI], *p* = 0.04), while female sex (HR 0.49 [0.33–0.45, 95% CI], *p* < 0.01) was associated with a lower risk of mortality. Thus, an increased urea-to-creatinine ratio is even stronger associated with long-term mortality than non-recovered AKI. Moreover, different from non-recovered AKI, the urea-to-creatinine ratio can already be determined upon presentation to the ED.

**Table 4:** Association of acute kidney injury and the urea-to-creatinine ratio with long-term all-cause mortality.

	Adj. HR (95% CI)^a^	*p*-value
Age (per 10 years)	1.32 (1.18–1.47)	< 0.01
Female sex	0.49 (0.33–0.45)	< 0.01
Malignancy	2.31 (1.61–3.32)	< 0.01
Acute kidney injury		
No AKI	*Reference*	
AKI, recovered	0.61 (0.33–1.13)	0.12
AKI, not recovered	1.86 (1.03–3.33)	0.04
Urea-to-creatinine ratio at ED		
Urea-to-creatinine ratio < 61	*Reference*	
Urea-to-creatinine ratio 61–84	0.79 (0.46–1.37)	0.40
Urea-to-creatinine ratio > 84	2.00 (1.37–2.91)	< 0.01

*HR* Hazard ratio, *95% CI* 95% Confidence interval, *AKI* Acute kidney injury.

^a^In addition to the factors shown in the table, co-morbidity (i.e. diabetes mellitus, cardiovascular disease, chronic kidney disease), SIRS score, septic shock and the relative change in creatinine and urea upon admission as compared to pre-existing values were entered in the multivariable forward: conditional Cox regression analysis. Model characteristics: *χ*^2^ 100.5, df 7, *p* < 0.01.

## Discussion

**Figure 4:**
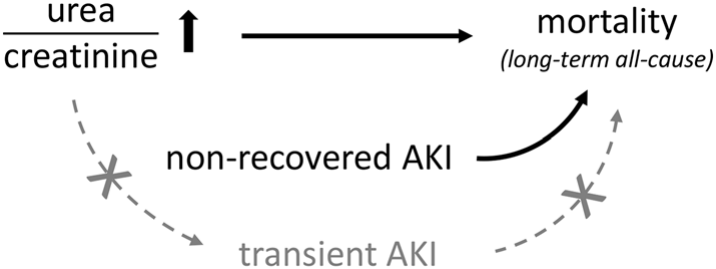
Relationship between the urea-to-creatinine ratio, different types of acute kidney injury and long-term mortality. An increased urea-to-creatinine ratio and non-recovered AKI were independent risk factors for long-term all-cause mortality. In contrast, an increased urea-to-creatinine ratio was not associated with transient AKI and transient AKI was not associated with increased long-term mortality. Black arrows denote an independent association, grey arrows denote a presumed association that was not confirmed in the current study.

Our study demonstrates that an increased urea-to-creatinine ratio upon hospitalization is an independent risk factor for long-term all-cause mortality in patients admitted to the hospital with infection. Also, transient AKI is not associated with long-term all-cause mortality among patients with (severe) infections, in contrast to non-recovered AKI at hospital discharge (Fig. 4). This is in line with previous studies in septic patients^12,24^, but was not yet known in patients with infection only. This means that the occurrence of AKI at admission does not predict long-term mortality at time of hospitalization as it is not known whether AKI will be transient or non-recovered. We hypothesized that a higher urea-to-creatinine ratio would be indicative of a relatively ‘benign’ course of AKI due to prerenal azotaemia^15–18^ and therefore associate with good prognosis and lower mortality. However, we did not find an association between the urea-to-creatinine ratio and AKI or a difference in the incidence of transient AKI after stratification of patients according to the urea-to-creatinine ratio. In contrast, we revealed an increased urea-to-creatinine ratio upon hospitalization to be a similar strong risk factor for long-term all-cause mortality as non-recovered AKI, independent of non-recovered AKI. Different from non-recovered AKI, however, the information needed to estimate the effect of the urea-to-creatinine ratio on long-term outcome after hospitalization is already present upon presentation to the ED. Finally, we identified SIRS score (specifically, tachycardia) to be associated with renal function recovery, while use of potassium-sparing or thiazide diuretics were associated with failure to recover from AKI. In contrast to the urea-to-creatinine ratio that was not associated with recovery of renal function, it seems that tachycardia might be a better indicator of pre-renal azotaemia and consequently associate with recovery from AKI.

Why the increased urea-to-creatinine ratio is such a strong risk factor for long-term all-cause mortality is not entirely clear. As patients with the highest urea-to-creatinine ratio also had the highest ratio in the year preceding ED presentation and at hospital discharge, the increased urea-to-creatinine ratio more likely reflects a chronic process associated with health, rather than acute hypovolemia leading to prerenal azotaemia. We postulate that the urea-to-creatinine ratio might serve as a biomarker indicative of frailty. Elderly are more susceptible to dehydration and consequently, an increased urea-to-creatinine-level, due to physical barriers to self-hydration, a decrease in thirst sensation, use of diuretics and co-morbidity that increases water loss, including diabetes mellitus^21,25^. However, as we do not have measures of the (change in) body weight or alternative measures of hydration status, we cannot conclude to what extent dehydration caused the increased urea-to-creatinine-level in our population. Additionally, higher serum urea levels due to a relative catabolic state secondary to low-grade inflammation^26^ or poor diet^19^, but also reduced serum creatinine levels secondary to low muscle mass or sarcopenia^27^ will increase the urea-to-creatinine ratio. Together, multiple factors associated with frailty can lead to a rise in the urea-to-creatinine-ratio, which may in turn identify older, frail people. Indeed, in our cohort of patients with non-severe infections and sepsis, patients with a higher urea-to-creatinine level were on average older and used more often diuretics, while co-morbidities were not different. Similar, Kirtane et al. demonstrated that patients hospitalized with acute coronary syndrome and elevated serum urea levels were on average older, had a higher prevalence of co-morbidities and an increased short and long-term mortality rate^28^. In extension of these populations, an increased urea-to-creatinine ratio is also associated with worsening of renal function and all-cause mortality after hospitalization for acute heart failure^19,20^ and long-term mortality among patients undergoing dialysis^21^. Thus, a higher urea-to-creatinine ratio might identify older, frail patients with an increased risk for long-term all-cause mortality after hospital discharge.

An increased urea-to-creatinine ratio likely reflects a chronic process associated with the health status of the patient, which could be used for risk stratification in patients with an infection. As such, the increased urea-to-creatinine ratio might be employed as a biomarker of frailty, preferably when combined with functional measurements (i.e. cognition, physical function) and other biomarkers (e.g. albumin, pre-albumin, weight change, total muscle mass). Identification of patients at risk for long-term mortality might allow for preventive measures to improve outcome after hospital discharge, like aftercare by a geriatric. Whether the urea-to-creatinine ratio will be of added benefit to support clinical decisions and whether long-term outcome of patients with an increased urea-to-creatinine ratio can be improved by (non-) pharmacological treatment remains to be studied.

Strengths of this study comprise its prospective design and near-complete four-year patient follow-up, with complete and comprehensive patient demography due to the municipal administration. We not only included patients with (severe) sepsis who might be in need of extensive treatment on the ICU, but also non-severe infections, which is why the current patient population is highly representative for the average ED population. To obtain data from a real-world situation we decided to base our inclusion on the clinician's suspicion of an infection, which may be suggested by the presence of fever, although we do realize that this strategy leaves room for debate about risk of selection bias. To reduce risk of selection bias, the treating physician was not involved in the study and researchers operated independent of the ED physicians.

Potential limitations of this study, however, are its single centre setting in a tertiary care hospital with referral of patients for specific specialist care. Another limitation is the retrospective retrieval of pre-existing creatinine levels of the patients, which was defined as the most recent measurement of creatinine level in either our centre or an external hospital. Although unlikely, between the moment the pre-existing creatinine was measured and the current event necessitating the ED visit, the patient may have developed chronic kidney disease, which could result in an overestimation of AKI in the study population. It should be noted that surgery, shock, intubation can affect recovery of renal function and long-term outcome as well. However, the incidence of critical illness in our population is low (3% ICU admissions) and no patient underwent surgery during hospitalization. This might affect generalization of our findings, although the current population is highly representative for the population of patients acutely admitted to the hospital with an infection.

## Conclusion

Our study shows that the urea-to-creatinine ratio is a strong indicator of long-term all-cause mortality, independent of the type of AKI. Since the course of renal function is not known at the time of hospitalization, renal function at admission is a less suitable predictor for mortality, in contrast to the urea-to-creatinine ratio.

Therefore the urea-to-creatinine ratio might be used to support clinical decision making by providing information on long-term mortality after hospitalization for an infection.

## Data Availability

The datasets generated during and/or analysed during the current study are available from the corresponding author on reasonable request.

## References

[1] Fleischmann C (2016). Assessment of global incidence and mortality of hospital-treated sepsis. Current estimates and Limitations. Am. J. Respir. Crit. Care Med..

[2] Kumar G (2011). Nationwide trends of severe sepsis in the 21st century (2000–2007). Chest.

[3] Mayr FB, Yende S, Angus DC (2014). Epidemiology of severe sepsis. Virulence.

[4] Angus DC (2001). Epidemiology of severe sepsis in the United States: Analysis of incidence, outcome, and associated costs of care. Crit. Care Med..

[5] Vincent JL (2006). Sepsis in European intensive care units: Results of the SOAP study. Crit. Care Med..

[6] Bagshaw SM (2009). Acute kidney injury in septic shock: Clinical outcomes and impact of duration of hypotension prior to initiation of antimicrobial therapy. Intensive Care Med..

[7] Uchino S (2005). Acute renal failure in critically ill patients: A multinational, multicenter study. JAMA.

[8] de Mendonca A (2000). Acute renal failure in the ICU: risk factors and outcome evaluated by the SOFA score. Intensive Care Med..

[9] Shum HP, Kong HH, Chan KC, Yan WW, Chan TM (2016). Septic acute kidney injury in critically ill patients—a single-center study on its incidence, clinical characteristics, and outcome predictors. Ren. Fail..

[10] Coca SG, Singanamala S, Parikh CR (2012). Chronic kidney disease after acute kidney injury: A systematic review and meta-analysis. Kidney Int..

[11] Soto K (2016). The risk of chronic kidney disease and mortality are increased after community-acquired acute kidney injury. Kidney Int..

[12] Fiorentino M (2018). Long-term survival in patients with septic acute kidney injury is strongly influenced by renal recovery. PLoS ONE.

[13] Mansfield KE (2018). Acute kidney injury and infections in patients taking antihypertensive drugs: A self-controlled case series analysis. Clin. Epidemiol..

[14] Murugan R (2010). Acute kidney injury in non-severe pneumonia is associated with an increased immune response and lower survival. Kidney Int..

[15] Nejat M (2012). Some biomarkers of acute kidney injury are increased in pre-renal acute injury. Kidney Int..

[16] Schrock JW, Glasenapp M, Drogell K (2012). Elevated blood urea nitrogen/creatinine ratio is associated with poor outcome in patients with ischemic stroke. Clin. Neurol. Neurosurg..

[17] Vullo-Navich K (1998). Comfort and incidence of abnormal serum sodium, BUN, creatinine and osmolality in dehydration of terminal illness. Am. J. Hosp. Palliat. Care.

[18] Aronson D (2008). Serum blood urea nitrogen and long-term mortality in acute ST-elevation myocardial infarction. Int. J. Cardiol..

[19] Brisco MA (2013). Blood urea nitrogen/creatinine ratio identifies a high-risk but potentially reversible form of renal dysfunction in patients with decompensated heart failure. Circ. Heart Fail..

[20] Murata A (2018). Relationship between blood urea nitrogen-to-creatinine ratio at hospital admission and long-term mortality in patients with acute decompensated heart failure. Heart Vessels.

[21] Inaguma D (2018). Ratio of blood urea nitrogen to serum creatinine at initiation of dialysis is associated with mortality: A multicenter prospective cohort study. Clin. Exp. Nephrol..

[22] von Elm E (2008). The strengthening the reporting of observational studies in epidemiology (STROBE) statement: guidelines for reporting observational studies. J. Clin. Epidemiol..

[23] Bone RC (1992). Definitions for sepsis and organ failure and guidelines for the use of innovative therapies in sepsis. The ACCP/SCCM Consensus Conference Committee. American College of Chest Physicians/Society of Critical Care Medicine. Chest.

[24] Mehta S (2018). The prognostic importance of duration of AKI: A systematic review and meta-analysis. BMC Nephrol..

[25] Kenney WL, Chiu P (2001). Influence of age on thirst and fluid intake. Med. Sci. Sports Exerc..

[26] Tanaka S (2017). Impact of blood urea nitrogen to creatinine ratio on mortality and morbidity in hemodialysis patients: The Q-Cohort Study. Sci. Rep..

[27] Baxmann AC (2008). Influence of muscle mass and physical activity on serum and urinary creatinine and serum cystatin C. Clin. J. Am. Soc. Nephrol..

[28] Kirtane AJ (2005). Serum blood urea nitrogen as an independent marker of subsequent mortality among patients with acute coronary syndromes and normal to mildly reduced glomerular filtration rates. J. Am. Coll. Cardiol..

